# Complete Genome Sequences of Two Shiga Toxin-Producing Escherichia coli O146:H10 Strains Recovered from a Foodborne Outbreak in China

**DOI:** 10.1128/MRA.00825-21

**Published:** 2021-10-28

**Authors:** Hui Huang, Yanwen Wang, Chunxu Wang, Yong Tang, Lili He, Jieyu Deng, Xianmin Pan

**Affiliations:** a Guilin Center for Disease Control and Prevention, Guilin, Guangxi, China; University of Maryland School of Medicine

## Abstract

Shiga toxin-producing Escherichia coli (STEC) is one of the primary pathogenic contaminants of foods, contributing to several foodborne outbreaks in recent years. Here, we report the complete genome sequences of two non-O157 STEC strains isolated from an outbreak of diarrhea in the city of Guilin, Guangxi Zhuang Autonomous Region, China.

## ANNOUNCEMENT

Shiga toxin-producing Escherichia coli (STEC) is a foodborne pathogen that can cause diarrhea and even life-threatening hemolytic uremic syndrome (HUS) in infected humans (1). In May 2019, an outbreak of diarrhea occurred in a middle school in Guilin, China. Two STEC strains (STEC865 and STEC866) were isolated from a stool sample from a diarrheal patient and a swab from a kitchen worker, respectively. The detection of STEC was conducted by using the national standard protocol for diagnostics for diarrheagenic Escherichia coli (GB 4789.6-2016). Briefly, all the samples were enriched with E. coli (EC) broth (Land Bridge, Beijing, China) at 37°C overnight. Four media were used to inoculate the enriched samples, including CHROMagar E. coli coliform (ECC) agar (Paris, France), MacConkey agar (Oxoid, Hampshire, UK), Rainbow agar O157 (RBA; Biolog Inc., Hayward, CA, USA) supplemented with 10 μg/ml novobiocin and 0.8 μg/ml potassium tellurite (modified) (RBA-NT), and CHROMagar STEC agar (CH-STEC); the samples were then incubated overnight at 37°C. Presumptive colonies were picked and tested for *stx* genes using the single-colony duplex PCR assay (2). Pulsed-field gel electrophoresis analysis revealed that the two isolates belonged to the same clone (2). This study was performed in strict accordance with human subject protection guidance provided by the Research Ethics Committee of Guilin Center for Disease Control and Prevention.

For DNA extraction, a single colony isolated from a Luria-Bertani agar plate was inoculated into lysogeny broth and cultivated overnight at 37°C with aeration. The Wizard genomic DNA purification kit (Promega, Madison, WI, USA) was used to prepare the DNA. Libraries for single-molecule real-time (SMRT) sequencing were constructed, with an insert size of 10 kb, using the SMRT bell template kit v. 1.0. SMRT sequencing was performed using the PacBio Sequel platform (Menlo Park, CA, USA). Short reads (1,000 bp or smaller) were removed using Filtlong v. 0.2.1 (https://github.com/rrwick/Filtlong). High-quality reads of STEC865 (117,783) and STEC866 (107,165) were obtained. The average lengths of the long reads of STEC865 and STEC866 were 7,826 bp and 7,942 bp, respectively. Libraries for Illumina were generated using the NEBNext Ultra DNA library prep kit following the manufacturer’s recommendations on the Illumina HiSeq 2500 platform with 2 × 150-bp paired-end reads. Low-quality reads showing quality values of less than 20 were removed using Sickle v. 1.33 (https://github.com/najoshi/sickle). Illumina reads were obtained for STEC865 (5,039,606) and STEC866 (3,333,334). Then, hybrid assemblies were generated using the SPAdes optimizer Unicycler v. 0.4.8 (3). Contigs less than 1,000 bp long or possessing k-mer coverage of less than 20 were excluded from the final assembly. QUAST v. 5.0.2 (4) was used to produce summary statistics for the assembly. Genome annotation was carried out using the National Center for Biotechnology Information (NCBI) Prokaryotic Genome Annotation Pipeline v. 4.12. BLASTN (https://blast.ncbi.nlm.nih.gov/Blast.cgi) was used to find sequence similarities.

Hybrid assembly of strains STEC865 and STEC866 generated a single circular chromosome with 2 circular plasmids for each strain. The plasmids were identical between these two isolates. The genomes of STEC865 and STEC866 were 4,944,083 bp (*N*_50_, 4,797,801 bp) and 4,944,441 bp (*N*_50_, 4,798,159 bp), respectively. The genome characteristics and accession numbers are presented in Table 1. Additionally, they were both identified as serotype O146:H10, sequence type 6198 (ST6198), and adhesin fimH type 33 using SerotypeFinder v. 2.0, MLST v. 2.0, and FimTyper v. 1.0, respectively (5–7). The molecular characteristics of the two strains suggest that these isolates might come from the same source; however, the source is unclear. Default parameter settings were used for all software unless otherwise indicated. The putative virulence factor genes were identified using the Virulence Factor Database (VFDB) (http://www.mgc.ac.cn/VFs/) (8) under the criteria of >80% identity and >60% coverage (Table 1).

**TABLE 1:** Genome statistics of the sequenced strains

Strain	Genetic element	GenBank accession no.	SRA accession no.	G+C content (%)	Genome size (bp)	Coverage (×)	No. of CDS^*a*^	No. of RNAs	Virulence factors^*b*^
STEC865	Chromosome	CP077649	SRX11598018, SRX11596556	50.7	4,797,801	280.8	4,660	112	CFA/I fimbriae, ECP, ELF, EaeH, HCP, type I fimbriae, EhaA, EhaB, UpaG adhesin, Ibes, EspL1, EspL4, EspR1, EspX1, EspX4, EspX5, ACE T6SS, hemolysin/cytolysin A, Shiga toxin
	p1_STEC865	CP077650		47.1	138,757	584.1	174	0	K88 fimbriae, vacuolating autotransporter, Ibes, AAI/SCI-II T6SS
	p2_STEC865	CP077651		46.5	7,525	7,828.2	14	0	
STEC866	Chromosome	CP077646	SRX11598019, SRX11596557	50.7	4,798,159	196.1	4,661	112	CFA/I fimbriae, ECP, ELF, EaeH, HCP, type I fimbriae, EhaA, EhaB, UpaG adhesin, Ibes, EspL1, EspL4, EspR1, EspX1, EspX4, EspX5, ACE T6SS, hemolysin/cytolysin A, Shiga toxin
	p1_STEC866	CP077647		47.1	138,757	251.5	175	0	K88 fimbriae, vacuolating autotransporter, Ibes, AAI/SCI-II T6SS
	p2_STEC866	CP077648		46.5	7,525	2,902.2	14	0	

a CDS, coding DNA sequences.

b ECP, E. coli common pilus; ELF, *E.coli* laminin-binding fimbriae; HCP, hemorrhagic E. coli pilus; Ibes, invasion of brain endothelial cells; CFA/I, colonization factor antigen I; ACE T6SS, ACE type VI secretion system; AAI/SCI-II T6SS, AAI/SCI-II type VI secretion system.

### Data availability.

The genome sequences have been deposited in GenBank under the BioProject number PRJNA739030. See Table 1 for the SRA and GenBank accession numbers.
